# (1*E*)-6-Meth­oxy-3,4-dihydro­naphthalen-1(2*H*)-one *O*-(*p*-tolyl­sulfon­yl)oxime

**DOI:** 10.1107/S1600536810039899

**Published:** 2010-10-09

**Authors:** Rong-Bi Han, Bo Zhang, Feng-Yu Piao

**Affiliations:** a Key Laboratory of Organism Functional Factors of the Changbai Moutain, Yanbian University, Ministry of Education, Yanji 133000, People’s Republic of China; b Institute of Chemical Technology of Yanbian University, Yanji 133000, People’s Republic of China; c Department of Chemistry, College of Science, Yanbian Universiy, Longjing, 133400, People’s Republic of China

## Abstract

In the title compound, C_18_H_19_NO_4_S, the two benzene rings form a dihedral angle of 68.37 (11)°. One of the C atoms of the fused ring bonded to the N atom displays positional disorder with site-occupation factors of 0.763 (7) and 0.237 (7) and the ring has an envelope conformation with the disordered C atoms located on opposite sides of the plane formed by the other atoms. In the crystal, inter­molecular C—H⋯O hydrogen bonds link the mol­ecules to form a two-dimensional supra­molecular network. The crystal structure is further stablized by weak inter­molecular C—H⋯π inter­actions.

## Related literature

The title compound has been used in our study (Byoung *et al.* 2000 ▶) of the effect of the reaction conditions on the Beckmanm rearrangement of 6-meth­oxy-3,4-dihydro­naphthalen-1(2*H*)-one oxime (Xiao *et al.*, 2007 ▶). For details of the synthesis, see Byoung *et al.* (2000 ▶). For a related structure, see Jin *et al.* (2010 ▶).
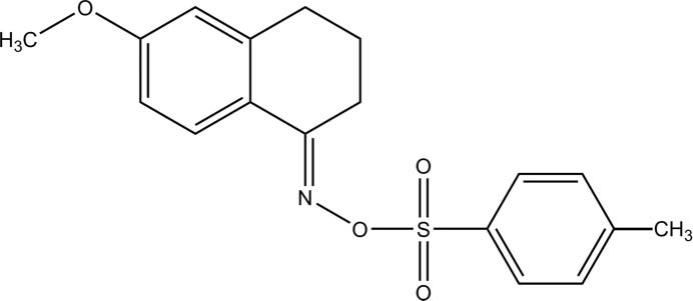


## Experimental

### 

#### Crystal data

C_18_H_19_NO_4_S
*M* *_r_* = 345.41
Monoclinic, 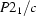

*a* = 13.478 (5) Å
*b* = 9.255 (5) Å
*c* = 17.707 (8) Å
β = 128.22 (3)°
*V* = 1735.3 (16) Å^3^
*Z* = 4
Mo *K*α radiation
μ = 0.21 mm^−1^
*T* = 290 K
0.12 × 0.11 × 0.10 mm

#### Data collection

Rigaku R-AXIS RAPID diffractometer
Absorption correction: multi-scan (*ABSCOR*; Higashi, 1995 ▶) *T*_min_ = 0.976, *T*_max_ = 0.980
16447 measured reflections
3939 independent reflections
3052 reflections with *I* > 2σ(*I*)
*R*_int_ = 0.029

#### Refinement

*R*[*F* ^2^ > 2σ(*F* ^2^)] = 0.044
*wR*(*F* ^2^) = 0.135
*S* = 1.01
3939 reflections
230 parameters
H-atom parameters constrained
Δρ_max_ = 0.44 e Å^−3^
Δρ_min_ = −0.34 e Å^−3^

### 

Data collection: *RAPID-AUTO* (Rigaku, 1998 ▶); cell refinement: *RAPID-AUTO*; data reduction: *CrystalStructure* (Molecular Structure Corporation & Rigaku, 2002 ▶); program(s) used to solve structure: *SHELXS97* (Sheldrick, 2008 ▶); program(s) used to refine structure: *SHELXL97* (Sheldrick, 2008 ▶); molecular graphics: *PLATON* (Spek, 2009 ▶); software used to prepare material for publication: *SHELXL97* (Sheldrick, 2008 ▶).

## Supplementary Material

Crystal structure: contains datablocks global, I. DOI: 10.1107/S1600536810039899/vm2048sup1.cif

Structure factors: contains datablocks I. DOI: 10.1107/S1600536810039899/vm2048Isup2.hkl

Additional supplementary materials:  crystallographic information; 3D view; checkCIF report

## Figures and Tables

**Table 1:** Hydrogen-bond geometry (Å, °) *Cg*1 is the centroid of the C12–C17 ring.

*D*—H⋯*A*	*D*—H	H⋯*A*	*D*⋯*A*	*D*—H⋯*A*
C6—H6⋯O1^i^	0.93	2.57	3.293 (3)	135
C10—H10*B*⋯O2^ii^	0.97	2.48	3.237 (5)	135
C15—H15⋯O1^iii^	0.93	2.68	3.430 (3)	139
C9—H9*B*⋯*Cg*1^iv^	0.97	2.85	3.750 (3)	156

Symmetry codes: (i) 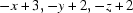; (ii) 
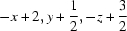
; (iii) 
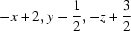
; (iv) 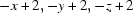.

**Fig. 1.**
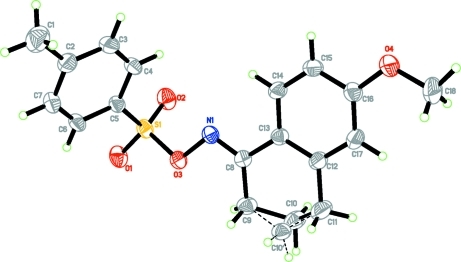
The molecular structure of the title compound, with the atom numbering. Displacement ellipsoids of non-H atoms are drawn at the 30% probalility level.

## supplementary crystallographic information

### Comment

Generally, 1,3,4,5-tetrahydro-7-methoxy-2*H*-1- benzazepin-2-one is obtained as major product from the Beckmanm rearrangement (BR) of 6-methoxy-3,4-dihydronaphthalen-1(2*H*)-one oxime (Xiao *et al.*, 2007). Recently, we have found that the product distribution of this BR greatly varied with reaction time and termperature (Byoung *et al.* 2000). We report here the crystal structure of the title comound, which was used in our attempts to study the effect of the reaction conditions on the ratio of the two isomers of product.

In the title compound, as shown in Fig. 1, all bond lengths and angles are normal and comparable with those reported for the related structure (Jin *et al.*, 2010). The disordered C10 and C10' atoms with site occupation factors of 0.76 and 0.24, respectively, lie at different sides of the plane defined by C8, C9, C11, C12 and C13. In the crystal, weak C—H···O hydrogen bonds (Table 1) link the molecules into a two-dimensional network. In additon, a C—H···π interaction between H9B and a neigboring benzene ring ocurs (H9B···*Cg*1^i^ = 2.846 (5) Å, *Cg*1 is the centroid of ring C12-C17, symmetry code i : 2 - *x*, 2 - *y*, 2 - z). The crystal structure is further stablized by Van der Waals' forces.

### Experimental

The title compound was prepared according to literature (Byoung *et al.* 2000) and single crystals suitable for X-ray diffraction were obtained from a solution of ethyl acetate by slow evaporation at room temperature.

### Refinement

All H atoms were placed in geometrically idealized positions and constrained to ride on their parent atoms, with distances C—H = 0.93, 0.96 and 0.97 Å for aryl, methyl and methylene H-atoms and *U*_iso_(H) = 1.5 (methyl) and 1.2 (the rest) *U*_eq_(C).

### Crystal data

**Table:**

C_18_H_19_NO_4_S	*F*(000) = 728
*M**_r_* = 345.41	*D*_x_ = 1.322 Mg m^−^^3^
Monoclinic, *P*2_1_/*c*	Mo *K*α radiation, λ = 0.71073 Å
Hall symbol: -P 2ybc	Cell parameters from 11474 reflections
*a* = 13.478 (5) Å	θ = 3.1–27.5°
*b* = 9.255 (5) Å	µ = 0.21 mm^−^^1^
*c* = 17.707 (8) Å	*T* = 290 K
β = 128.22 (3)°	Block, colorless
*V* = 1735.3 (16) Å^3^	0.12 × 0.11 × 0.10 mm
*Z* = 4	

### Data collection

**Table:**

Rigaku R-AXIS RAPID diffractometer	3939 independent reflections
Radiation source: fine-focus sealed tube	3052 reflections with *I* > 2σ(*I*)
graphite	*R*_int_ = 0.029
ω scans	θ_max_ = 27.5°, θ_min_ = 3.1°
Absorption correction: multi-scan (*ABSCOR*; Higashi, 1995)	*h* = −17→17
*T*_min_ = 0.976, *T*_max_ = 0.980	*k* = −11→11
16447 measured reflections	*l* = −22→21

### Refinement

**Table:**

Refinement on *F*^2^	Secondary atom site location: difference Fourier map
Least-squares matrix: full	Hydrogen site location: inferred from neighbouring sites
*R*[*F*^2^ > 2σ(*F*^2^)] = 0.044	H-atom parameters constrained
*wR*(*F*^2^) = 0.135	*w* = 1/[σ^2^(*F*_o_^2^) + (0.0686*P*)^2^ + 0.4858*P*] where *P* = (*F*_o_^2^ + 2*F*_c_^2^)/3
*S* = 1.01	(Δ/σ)_max_ = 0.001
3939 reflections	Δρ_max_ = 0.44 e Å^−^^3^
230 parameters	Δρ_min_ = −0.34 e Å^−^^3^
0 restraints	Extinction correction: *SHELXL97* (Sheldrick, 2008), Fc^*^=kFc[1+0.001xFc^2^λ^3^/sin(2θ)]^-1/4^
Primary atom site location: structure-invariant direct methods	Extinction coefficient: 0.069 (4)

### Special details

**Table:**

Experimental. (See detailed section in the paper)
Geometry. All e.s.d.'s (except the e.s.d. in the dihedral angle between two l.s. planes) are estimated using the full covariance matrix. The cell e.s.d.'s are taken into account individually in the estimation of e.s.d.'s in distances, angles and torsion angles; correlations between e.s.d.'s in cell parameters are only used when they are defined by crystal symmetry. An approximate (isotropic) treatment of cell e.s.d.'s is used for estimating e.s.d.'s involving l.s. planes.
Refinement. Refinement of *F*^2^ against ALL reflections. The weighted *R*-factor *wR* and goodness of fit *S* are based on *F*^2^, conventional *R*-factors *R* are based on *F*, with *F* set to zero for negative *F*^2^. The threshold expression of *F*^2^ > σ(*F*^2^) is used only for calculating *R*-factors(gt) *etc*. and is not relevant to the choice of reflections for refinement. *R*-factors based on *F*^2^ are statistically about twice as large as those based on *F*, and *R*- factors based on ALL data will be even larger.

### Fractional atomic coordinates and isotropic or equivalent isotropic displacement parameters (Å^2^)

**Table:**

	*x*	*y*	*z*	*U*_iso_*/*U*_eq_	Occ. (<1)
S1	1.21154 (4)	1.01704 (6)	0.84699 (3)	0.05339 (18)	
O1	1.32298 (13)	1.09992 (16)	0.88664 (11)	0.0688 (4)	
O2	1.11691 (15)	1.01052 (18)	0.74596 (10)	0.0720 (4)	
O3	1.15381 (12)	1.09228 (14)	0.89346 (9)	0.0557 (3)	
O4	0.53547 (14)	0.81979 (17)	0.77887 (12)	0.0738 (4)	
N1	1.03738 (14)	1.01680 (16)	0.85957 (11)	0.0510 (4)	
C1	1.3561 (3)	0.4210 (3)	1.0232 (2)	0.0928 (8)	
H1A	1.3260	0.3480	0.9750	0.139*	
H1B	1.3191	0.4067	1.0547	0.139*	
H1C	1.4464	0.4148	1.0696	0.139*	
C2	1.31973 (19)	0.5680 (2)	0.97663 (15)	0.0600 (5)	
C3	1.19864 (19)	0.5964 (2)	0.89321 (15)	0.0624 (5)	
H3	1.1395	0.5222	0.8642	0.075*	
C4	1.16441 (17)	0.7318 (2)	0.85263 (13)	0.0562 (5)	
H4	1.0827	0.7489	0.7971	0.067*	
C5	1.25256 (15)	0.8425 (2)	0.89508 (12)	0.0475 (4)	
C6	1.37496 (16)	0.8166 (2)	0.97757 (13)	0.0534 (4)	
H6	1.4345	0.8905	1.0058	0.064*	
C7	1.40688 (18)	0.6800 (2)	1.01695 (14)	0.0613 (5)	
H7	1.4889	0.6625	1.0719	0.074*	
C8	0.97831 (16)	1.08889 (18)	0.88218 (11)	0.0450 (4)	
C9	1.0207 (2)	1.2307 (2)	0.93435 (15)	0.0603 (5)	
H9A	1.0587	1.2886	0.9126	0.072*	
H9B	1.0842	1.2136	1.0027	0.072*	
C10	0.9081 (4)	1.3146 (3)	0.9165 (3)	0.0698 (12)	0.763 (7)
H10A	0.9403	1.3983	0.9584	0.084*	0.763 (7)
H10B	0.8536	1.3488	0.8506	0.084*	0.763 (7)
C11	0.8345 (3)	1.2276 (3)	0.9335 (2)	0.0809 (7)	
H11A	0.7615	1.2826	0.9155	0.097*	
H11B	0.8851	1.2063	1.0017	0.097*	
C12	0.79064 (18)	1.08776 (19)	0.87810 (13)	0.0529 (4)	
C10'	0.9604 (8)	1.2634 (9)	0.9825 (7)	0.053 (3)	0.237 (7)
H10C	1.0092	1.2141	1.0441	0.064*	0.237 (7)
H10D	0.9688	1.3662	0.9958	0.064*	0.237 (7)
C13	0.86031 (17)	1.02224 (18)	0.85398 (12)	0.0454 (4)	
C14	0.81694 (19)	0.8901 (2)	0.80425 (15)	0.0574 (5)	
H14	0.8619	0.8455	0.7868	0.069*	
C15	0.70982 (19)	0.8259 (2)	0.78110 (14)	0.0593 (5)	
H15	0.6834	0.7377	0.7491	0.071*	
C16	0.64081 (18)	0.8922 (2)	0.80522 (14)	0.0549 (5)	
C17	0.6813 (2)	1.0222 (2)	0.85367 (16)	0.0616 (5)	
H17	0.6352	1.0664	0.8702	0.074*	
C18	0.4673 (3)	0.8776 (3)	0.8090 (2)	0.0938 (8)	
H18A	0.4301	0.9682	0.7773	0.141*	
H18B	0.5238	0.8923	0.8773	0.141*	
H18C	0.4020	0.8112	0.7928	0.141*	

### Atomic displacement parameters (Å^2^)

**Table:**

	*U*^11^	*U*^22^	*U*^33^	*U*^12^	*U*^13^	*U*^23^
S1	0.0438 (3)	0.0658 (3)	0.0536 (3)	−0.0040 (2)	0.0316 (2)	0.0018 (2)
O1	0.0533 (8)	0.0715 (9)	0.0878 (10)	−0.0104 (7)	0.0468 (8)	0.0057 (7)
O2	0.0609 (9)	0.0995 (12)	0.0511 (8)	0.0048 (8)	0.0324 (7)	0.0083 (7)
O3	0.0463 (7)	0.0579 (8)	0.0656 (8)	−0.0083 (6)	0.0360 (6)	−0.0078 (6)
O4	0.0601 (9)	0.0825 (10)	0.0925 (11)	−0.0191 (8)	0.0540 (8)	−0.0229 (8)
N1	0.0445 (8)	0.0534 (8)	0.0581 (9)	−0.0071 (6)	0.0333 (7)	−0.0044 (7)
C1	0.0898 (18)	0.0653 (15)	0.120 (2)	0.0104 (13)	0.0630 (17)	0.0081 (14)
C2	0.0582 (11)	0.0550 (11)	0.0729 (12)	−0.0001 (9)	0.0437 (10)	−0.0079 (9)
C3	0.0536 (11)	0.0591 (12)	0.0713 (12)	−0.0154 (9)	0.0371 (10)	−0.0208 (9)
C4	0.0404 (9)	0.0651 (12)	0.0523 (10)	−0.0100 (8)	0.0234 (8)	−0.0147 (8)
C5	0.0384 (8)	0.0581 (10)	0.0473 (9)	−0.0061 (7)	0.0271 (7)	−0.0084 (7)
C6	0.0372 (9)	0.0621 (11)	0.0547 (10)	−0.0100 (8)	0.0253 (8)	−0.0095 (8)
C7	0.0413 (10)	0.0717 (13)	0.0605 (11)	0.0022 (9)	0.0263 (9)	−0.0019 (9)
C8	0.0485 (9)	0.0461 (9)	0.0425 (8)	0.0004 (7)	0.0292 (7)	0.0029 (7)
C9	0.0692 (12)	0.0513 (10)	0.0713 (12)	−0.0148 (9)	0.0490 (11)	−0.0123 (9)
C10	0.093 (2)	0.0427 (15)	0.097 (3)	−0.0078 (15)	0.070 (2)	−0.0106 (16)
C11	0.0880 (17)	0.0619 (13)	0.1167 (19)	−0.0127 (12)	0.0753 (16)	−0.0318 (13)
C12	0.0575 (11)	0.0479 (10)	0.0607 (10)	−0.0007 (8)	0.0404 (9)	−0.0056 (8)
C10'	0.059 (5)	0.041 (4)	0.055 (5)	−0.001 (3)	0.033 (4)	−0.005 (4)
C13	0.0498 (9)	0.0462 (9)	0.0452 (8)	−0.0020 (7)	0.0318 (8)	−0.0022 (7)
C14	0.0605 (11)	0.0594 (11)	0.0698 (12)	−0.0097 (9)	0.0490 (10)	−0.0185 (9)
C15	0.0619 (12)	0.0582 (11)	0.0661 (11)	−0.0140 (9)	0.0438 (10)	−0.0210 (9)
C16	0.0505 (10)	0.0610 (11)	0.0583 (10)	−0.0078 (8)	0.0362 (9)	−0.0062 (8)
C17	0.0608 (12)	0.0628 (12)	0.0780 (13)	−0.0003 (9)	0.0513 (11)	−0.0110 (10)
C18	0.0728 (16)	0.0988 (19)	0.140 (2)	−0.0144 (14)	0.0808 (18)	−0.0213 (17)

### Geometric parameters (Å, °)

**Table:**

S1—O2	1.4169 (17)	C9—C10	1.553 (4)
S1—O1	1.4257 (15)	C9—H9A	0.9700
S1—O3	1.5997 (14)	C9—H9B	0.9700
S1—C5	1.748 (2)	C10—C11	1.446 (4)
O3—N1	1.465 (2)	C10—H10A	0.9700
O4—C16	1.365 (2)	C10—H10B	0.9700
O4—C18	1.422 (3)	C10—H10D	1.2028
N1—C8	1.278 (2)	C11—C12	1.507 (3)
C1—C2	1.507 (3)	C11—H11A	0.9700
C1—H1A	0.9600	C11—H11B	0.9700
C1—H1B	0.9600	C12—C13	1.388 (2)
C1—H1C	0.9600	C12—C17	1.393 (3)
C2—C7	1.388 (3)	C10'—H10C	0.9700
C2—C3	1.390 (3)	C10'—H10D	0.9700
C3—C4	1.374 (3)	C13—C14	1.406 (3)
C3—H3	0.9300	C14—C15	1.368 (3)
C4—C5	1.387 (3)	C14—H14	0.9300
C4—H4	0.9300	C15—C16	1.384 (3)
C5—C6	1.390 (3)	C15—H15	0.9300
C6—C7	1.378 (3)	C16—C17	1.379 (3)
C6—H6	0.9300	C17—H17	0.9300
C7—H7	0.9300	C18—H18A	0.9600
C8—C13	1.475 (2)	C18—H18B	0.9600
C8—C9	1.499 (3)	C18—H18C	0.9600
			
O2—S1—O1	119.72 (10)	C11—C10—C9	112.9 (3)
O2—S1—O3	108.89 (9)	C11—C10—H10A	109.0
O1—S1—O3	102.24 (9)	C9—C10—H10A	109.0
O2—S1—C5	110.00 (9)	C11—C10—H10B	109.0
O1—S1—C5	109.74 (9)	C9—C10—H10B	109.0
O3—S1—C5	105.04 (8)	H10A—C10—H10B	107.8
N1—O3—S1	108.69 (10)	C11—C10—H10D	92.1
C16—O4—C18	117.66 (18)	C9—C10—H10D	95.1
C8—N1—O3	109.86 (14)	H10A—C10—H10D	29.6
C2—C1—H1A	109.5	H10B—C10—H10D	137.4
C2—C1—H1B	109.5	C10—C11—C12	112.7 (2)
H1A—C1—H1B	109.5	C10—C11—H11A	109.1
C2—C1—H1C	109.5	C12—C11—H11A	109.1
H1A—C1—H1C	109.5	C10—C11—H11B	109.1
H1B—C1—H1C	109.5	C12—C11—H11B	109.1
C7—C2—C3	117.94 (19)	H11A—C11—H11B	107.8
C7—C2—C1	120.5 (2)	C13—C12—C17	120.08 (17)
C3—C2—C1	121.5 (2)	C13—C12—C11	120.54 (18)
C4—C3—C2	121.47 (18)	C17—C12—C11	119.35 (18)
C4—C3—H3	119.3	H10C—C10'—H10D	107.1
C2—C3—H3	119.3	C12—C13—C14	118.31 (17)
C3—C4—C5	119.52 (18)	C12—C13—C8	120.39 (16)
C3—C4—H4	120.2	C14—C13—C8	121.28 (16)
C5—C4—H4	120.2	C15—C14—C13	121.21 (17)
C4—C5—C6	120.27 (18)	C15—C14—H14	119.4
C4—C5—S1	120.82 (14)	C13—C14—H14	119.4
C6—C5—S1	118.91 (14)	C14—C15—C16	120.07 (18)
C7—C6—C5	119.09 (17)	C14—C15—H15	120.0
C7—C6—H6	120.5	C16—C15—H15	120.0
C5—C6—H6	120.5	O4—C16—C17	124.63 (18)
C6—C7—C2	121.69 (18)	O4—C16—C15	115.63 (17)
C6—C7—H7	119.2	C17—C16—C15	119.73 (18)
C2—C7—H7	119.2	C16—C17—C12	120.59 (18)
N1—C8—C13	115.19 (16)	C16—C17—H17	119.7
N1—C8—C9	125.19 (17)	C12—C17—H17	119.7
C13—C8—C9	119.62 (15)	O4—C18—H18A	109.5
C8—C9—C10	111.14 (19)	O4—C18—H18B	109.5
C8—C9—H9A	109.4	H18A—C18—H18B	109.5
C10—C9—H9A	109.4	O4—C18—H18C	109.5
C8—C9—H9B	109.4	H18A—C18—H18C	109.5
C10—C9—H9B	109.4	H18B—C18—H18C	109.5
H9A—C9—H9B	108.0		
			
O2—S1—O3—N1	52.91 (14)	C8—C9—C10—C11	50.4 (4)
O1—S1—O3—N1	−179.47 (11)	C9—C10—C11—C12	−53.4 (4)
C5—S1—O3—N1	−64.88 (12)	C10—C11—C12—C13	28.5 (4)
S1—O3—N1—C8	−169.09 (12)	C10—C11—C12—C17	−153.5 (3)
C7—C2—C3—C4	−1.6 (3)	C17—C12—C13—C14	0.5 (3)
C1—C2—C3—C4	177.8 (2)	C11—C12—C13—C14	178.5 (2)
C2—C3—C4—C5	0.6 (3)	C17—C12—C13—C8	−178.15 (17)
C3—C4—C5—C6	0.6 (3)	C11—C12—C13—C8	−0.1 (3)
C3—C4—C5—S1	−178.62 (15)	N1—C8—C13—C12	177.43 (16)
O2—S1—C5—C4	−31.08 (18)	C9—C8—C13—C12	−2.0 (3)
O1—S1—C5—C4	−164.80 (15)	N1—C8—C13—C14	−1.1 (3)
O3—S1—C5—C4	85.95 (16)	C9—C8—C13—C14	179.43 (18)
O2—S1—C5—C6	149.71 (15)	C12—C13—C14—C15	−0.9 (3)
O1—S1—C5—C6	16.00 (17)	C8—C13—C14—C15	177.70 (18)
O3—S1—C5—C6	−93.26 (16)	C13—C14—C15—C16	1.1 (3)
C4—C5—C6—C7	−0.7 (3)	C18—O4—C16—C17	−4.3 (3)
S1—C5—C6—C7	178.53 (15)	C18—O4—C16—C15	174.9 (2)
C5—C6—C7—C2	−0.4 (3)	C14—C15—C16—O4	179.88 (18)
C3—C2—C7—C6	1.5 (3)	C14—C15—C16—C17	−0.8 (3)
C1—C2—C7—C6	−177.9 (2)	O4—C16—C17—C12	179.6 (2)
O3—N1—C8—C13	−178.59 (13)	C15—C16—C17—C12	0.4 (3)
O3—N1—C8—C9	0.8 (2)	C13—C12—C17—C16	−0.2 (3)
N1—C8—C9—C10	158.5 (2)	C11—C12—C17—C16	−178.3 (2)
C13—C8—C9—C10	−22.2 (3)		

### Hydrogen-bond geometry (Å, °)

**Table:**

*Cg*1 is the centroid of the C12–C17 ring.

**Table:**

*D*—H···*A*	*D*—H	H···*A*	*D*···*A*	*D*—H···*A*
C6—H6···O1^i^	0.93	2.57	3.293 (3)	135
C10—H10B···O2^ii^	0.97	2.48	3.237 (5)	135
C15—H15···O1^iii^	0.93	2.68	3.430 (3)	139
C9—H9B···Cg1^iv^	0.97	2.85	3.750 (3)	156

Symmetry codes: (i) −*x*+3, −*y*+2, −*z*+2; (ii) −*x*+2, *y*+1/2, −*z*+3/2; (iii) −*x*+2, *y*−1/2, −*z*+3/2; (iv) −*x*+2, −*y*+2, −*z*+2.
